# Asiatic Acid, a Natural Compound that Exerts Beneficial Effects on the Cystometric and Biochemical Parameters in the Retinyl Acetate-Induced Model of Detrusor Overactivity

**DOI:** 10.3389/fphar.2020.574108

**Published:** 2021-01-29

**Authors:** Andrzej Wróbel, Anna Serefko, Aleksandra Szopa, Ewa Poleszak

**Affiliations:** ^1^ Second Department of Gynecology, Medical University of Lublin, Lublin, Poland; ^2^ Chair and Department of Applied and Social Pharmacy, Laboratory of Preclinical Testing, Medical University of Lublin, Lublin, Poland

**Keywords:** asiatic acid, detrusor overactivity, retinyl acetate, rats, oxidative stress

## Abstract

Scientists have been constantly looking for new synthetic and natural compounds that could have beneficial effects in bladder overactivity. Our attention was drawn by asiatic acid that influences a number of molecules and signaling pathways relevant for the proper functioning of the urinary tracts in humans. In the present project we wanted to check whether asiatic acid would have positive effects in the confirmed animal model of detrusor overactivity (DO) and whether it would affect the bladder blood flow, urothelium thickness, inflammatory and oxidative stress markers, neurotrophic and growth factors, and other parameters important for the activity of the urinary bladder. The outcomes of our study showed that a 14-day administration of asiatic acid (30 mg/kg/day) by oral gavage normalizes the cystometric parameters corresponding to DO and reduces the accompanying oxidative stress (measured by the levels of malondialdehyde–61,344 ± 24,908 pg/ml vs. 33,668 ± 5,071 pg/ml, 3-nitrotyrosine–64,615 ± 25,433 pg/ml vs. 6,563 ± 1,736 pg/ml, and NOS2–2,506 ± 411.7 vs. 3,824 ± 470.1 pg/ml). Moreover, it decreases the urinary secretion of neurotrophins (BDNF–304.4 ± 33.21 pg/ml vs. 119.3 ± 11.49 pg/ml and NGF–205.5 ± 18.50 vs. 109.7 ± 15.94 pg/ml) and prevents the changes in a range of biomarkers indicating the dysfunction of the urinary bladder, CGRP (421.1 ± 56.64 vs. 108.1 ± 11.73 pg/ml), E-Cadherin (773.5 ± 177.5 pg/ml vs. 1,560 ± 154.5 pg/ml), OCT3 (3,943 ± 814.6 vs. 1,018 ± 97.07 pg/ml), SNAP-23 (6,763 ± 808.9 pg/ml vs. 3,455 ± 554.5 pg/ml), SNAP-25 (2,038 ± 162.7 pg/ml vs. 833.3 ± 65.48), substance P (171.7 ± 16.86 pg/ml vs. 65.07 ± 8.250 pg/ml), SV2A (1,927 ± 175.3 pg/ml vs. 1,154 ± 254.9 pg/ml), tight junction protein 1 (360.1 ± 95.05 pg/ml vs. 563.4 ± 65.43 pg/ml), VAChT (16,470 ± 2,419 pg/ml vs. 7,072 ± 1,339 pg/ml), VEGFA (318.3 ± 37.89 pg/ml vs. 201.5 ± 22.91 pg/ml). The mentioned parameters are associated with smooth muscle contractions, urothelial barrier, transportation and release of transmitters, or bladder compensation. Thus, the presented findings allow to suggest a possible future role of asiatic acid in the prevention of conditions accompanied by DO, such as overactive bladder.

## Introduction

Overactive bladder (OAB) is described as a chronic condition characterized by urinary urgency, with or without urge incontinence, usually with frequency and nocturia (Abrams et al., 2002). Due to these symptoms OAB patients may also feel frustration, embarrassment, and a high level of anxiety which significantly worsen their quality of life and limit social activities. Though the results of studies estimating the prevalence of OAB vary significantly (mostly due to a non-uniform methodology), this syndrome affects up to 16% of people in the developed countries. Given the fact that the incidence of OAB increases with age and that the elderly population is growing in Western countries, it is highly possible that bladder overactivity will be a very important public health problem in the nearest future (Eapen and Radomski, 2016). The etiology of OAB has not been fully discovered yet. There are several hypotheses related to the pathophysiology of this syndrome and to the dysfunction of the detrusor muscle which frequently accompanies other symptoms of OAB (Leron et al., 2018). Depending on the study design, about 60% (up to 90%) of patients with OAB are also diagnosed with detrusor overactivity (DO) (Hashim and Abrams, 2006; Al-Ghazo et al., 2011). Attenuation of the central inhibitory pathways, intensification of afferent impulses from the bladder (de Groat, 1997), sensitization of the detrusor muscle to cholinergic stimulation (Brading, 2006), changes in phasic activity of the urinary bladder (Drake et al., 2001), “afferent noise” produced by spontaneous activity of the detrusor muscle during the filling phase (Andersson, 2010), or imbalance between the excitatory and inhibitory autonomic modulation of the activity of the detrusor muscle (Wein and Rackley, 2006) may be responsible for the development of OAB/DO.

Pharmacological management of OAB is highly challenging due to a shortage of effective drugs. It relies predominantly on administration of the anticholinergic agents. They attenuate contractions of the detrusor muscle and inhibit the activity of afferent nerves (Robinson and Cardozo, 2012). Thus, they seem to influence both phases of the micturition reflex. However, therapy with anticholinergic drugs is not successful in all patients and it is frequently associated with bothersome side effects (e.g., constipation, dry mouth, blurred vision, somnolence, confusion) that may lead to discontinuation of the introduced treatment. Furthermore, antimuscarinics do not act immediately–improvement of symptoms appears gradually, which can also be a factor negatively affecting patient’s compliance (Leron et al., 2018). According to the literature data (Sexton et al., 2011), up to 43–83% of patients may decide to stop taking their anticholinergic drugs within the first 30 days of treatment. As the second-line therapy for OAB, β_3_-adrenoceptor agonists (mirabegron, vibegron) and onabotulinumtoxin A are used (Leron et al., 2018).

Since the treatment options are scarce, scientists are constantly looking for new synthetic and natural compounds that could have beneficial effects in bladder overactivity. Investigational therapies are mainly based on interactions with receptors and neurotransmitters that modulate the micturition reflex. Thus, our particular attention was drawn by asiatic acid. It is one of the pentacyclic triterpenoids of *Centella asiatica* (so-called gotu kola), i.e. a medicinal herb belonging to the Apiaceae family. Both asiatic acid and its derivatives seem to be promising agents for the future treatment of different diseases (Lv et al., 2018). A wide range of preclinical studies have revealed the anti-inflammatory (Dong et al., 2017), antioxidant (Qi et al., 2017), antidiabetic (Zhang et al., 2009; Frias et al., 2011), hepatoprotective (Gao et al., 2017), neuroprotective (Jiang et al., 2016), antiobesity (Uddandrao et al., 2019), and antimicrobial (Liu et al., 2015) properties of asiatic acid. Furthermore, diverse research teams have suggested that asiatic acid could have beneficial effects in the management and/or prevention of cancers (Islam et al., 2019), epilepsy (Wang et al., 2018), Parkinson’s (Ding et al., 2018) and Alzheimer’s (Ahmad et al., 2019) diseases, neuropathy (Lou et al., 2018), osteoporosis (Huang et al., 2019), metabolic syndrome (Sharma et al., 2018), and other conditions. This multidirectional biological activity of asiatic acid is due to its influence on about 100 different molecular targets *via* direct or indirect interactions (i.e., an impact on gene expression and/or signaling pathways). A great number of these molecules and signaling pathways is relevant for the proper functioning of the urinary tracts in humans, including pro- and anti-inflammatory cytokines (IL-1, IL-4, IL-5, IL-6, IL-10, TNF-α), chemokines (CXCL1, MCP-1, MIP-2), growth factors (BDNF, CTGF, FGF, TGF, VEGF), enzymes (AChE, BChE, COX-2, iNOS, NOx, MAPK, MPO), signaling (AMPK/CREB, ERK1/2, PI3K/Akt) and adhesion (ICAM-1, VCAM-1) proteins, receptors (μ-opioid, GABA_A_, GABA_B_, TLRs) as well as transcription factors (NF-kB, STATs) (for review see Nagoor Meeran et al., 2018).

So far, there are no reports related to the possible use of asiatic acid in medical conditions with unstable bladder contractions, including OAB and DO. However, in the traditional medicine gotu kola is considered as an adjuvant herb for urinary tract infections and stress incontinence (Tilgner, 2000). It is believed that *C. asiatica* supports functioning of the urinary tracts and its organs by the tonic activity toward connective tissues and the nervous system as well as by strengthening ligaments and fascias (DiPasquale, 2008). Therefore, in the presented studies we decided to investigate the effects of asiatic acid in one of the confirmed animal models of DO, i.e. the retinyl acetate one. Results of our previous projects (Wróbel et al., 2015; Wróbel and Rechberger, 2017) revealed that a single transient instillation of 0.75% retinyl acetate solution induces DO without producing inflammatory lesions in the urothelium in conscious female rats. By acting on the vanilloid receptor 1 (TRPV1) retinyl acetate stimulates nociceptive afferent C-fibres which leads to sensory hypersensitivity–a potential cause of urinary urgency. The applied model of DO is sensitive to oxybutynin chloride which is an anticholinergic agent used in the treatment of OAB (Wróbel and Rechberger, 2017). Regarding the subjective nature of OAB symptoms, it’s almost impossible to design a reliable animal model of this disease. Therefore most pre-clinical studies are based on more objective models of DO, i.e. the urodynamic observation of involuntary bladder contractions during the filling phase which can be automatically measured by cystometry. For sure, such an approach entails some risk of misinterpreting the obtained results and underlying processes since OAB and DO cannot be considered as interchangeable. However, at least for the time being, animal models of the induced DO seem to be the best research tool for determination of the potential of novel agents in the treatment of OAB (Parsons and Drake, 2011). In the present study we also wanted to evaluate the influence of asiatic acid on the bladder blood flow, urothelium thickness, inflammatory markers, oxidative stress markers, neurotrophic and growth factors, and other parameters which levels can be altered in diseases of the urinary bladder.

As the experimental subjects we used Wistar female rats having in mind that OAB is more common for women than for men (Eapen and Radomski, 2016). Most probably, estrogens are partially responsible for such a phenomena. Estrogen receptors are present in the urethra, urothelium, and the bladder detrusor muscle. Estrogens influence the cholinergic innervations that control functions of the urinary bladder, whereas estrogen deficiency is a risk factor of OAB development (Robinson and Cardozo, 2003; Parekh et al., 2004; Yoshida et al., 2007). Since animals have different cystometry outcomes in wakefulness than in a condition of slight/deep anesthesia, we decided to keep the tested rats in the conscious state while performing the measurements in order to eliminate the influence of anesthetic drugs on micturition (Andersson et al., 2011).

Keeping the above in mind, the objective of the current exploratory research was to evaluate the effectiveness of asiatic acid in the animal model of retinyl acetate-induced DO. Moreover, the influence of this natural compound on OAB-specific biomarkers was assessed.

## Materials and Methods

All described experiments were approved by the Local Ethics Committee and they were performed in accordance with European law related to the experimental studies on animal models (i.e., the ARRIVE guidelines and the EU Directive 2010/63/EU).

### Animals

All experimental procedures were carried out on naive female Wistar rats (age: 3 weeks; initial weight: ca. 250 g) purchased from a licensed breeder (The Experimental Medicine Center of the Medical University of Lublin, Poland).They were individually kept in metabolic cages with free access to water and food. The cages were placed in environmentally controlled rooms with following conditions: natural light/dark cycle, temperature of 22–23°C, relative humidity of 45–55%. Animals were randomly assigned to one of the four experimental groups (n = 15 rats/group):

1. given vehicle I (once) plus vehicle II (for 14 days), i.e. the control group
2. given retinyl acetate (0.75% solution, once) plus vehicle II (for 14 days)
3. given vehicle I (once) plus asiatic acid (30 mg/kg/day for 14 days)
4. given retinyl acetate (0.75% solution, once) plus asiatic acid (30 mg/kg/day for 14 days).

### Drugs

The following drugs were used in the study: retinyl acetate (purity: ≥90%, company: Sigma-Aldrich, Poznań, Poland) and asiatic acid (trans-(1R, 9S)-8-Methylene-4,11,11-trimethylbicyclo [7.2.0] undec-ene; Catalog No.: BP0203, purity: 98%, company: Biopurify Phytochemicals Ltd., China). Retinyl acetate was diluted to 0.75% solution with a mixture of polysorbate 80 and saline and it was given only once *via* an intravesical instillation. The final concentration of the nonionic surfactant was 0.005 mM. Then, a 14-day treatment with asiatic acid (30 mg/kg/day) was started. Asiatic acid was dissolved in 1% DMSO solution and it was administered by oral gavage. The control animals were given a volume-matched dose of the vehicles, i.e. vehicle I–an intravesical instillation of the polysorbate 80-saline mixture and/or vehicle II–1% DMSO solution *via* oral gavage. Both doses of the administered drugs and the treatment schedules were chosen on the basis of our previous projects (e.g., Wróbel et al., 2015; Wróbel and Rechberger, 2017) and they were confirmed in preliminary studies carried out in our lab.

### Surgical procedures

The details of the performed surgical procedure we described before (e.g., Wróbel et al., 2015; Wróbel and Rechberger, 2017). It was carried out under anesthesia that does not abolish the micturition reflex in female rats (Cannon and Damaser, 2001), i.e. the intraperitoneal administration of ketamine hydrochloride (75 mg/kg; Ketanest, Pfizer) and xylazine (15 mg/kg; Sedazin, Biowet). Briefly, at first, the urinary bladder was catheterized with a polyethylene catheter through the external urethral meatus. The bladder was emptied and the retinyl acetate solution or vehicle was installed until the intravesical pressure extended to 10 cm H_2_O. Five minutes later, the solution was removed and the bladder was washed mildly three times with physiological saline. After that, the urethral catheter was removed and the 10-mm abdominal midline vertical incision was performed. A double lumen catheter was inserted through the apex of the bladder dome and fixed with a 6–0 suture. Lastly, abdominal wall was sutured and closed in layers.

### Cystometric Analyses and Assessment of the Bladder Blood Flow

Cystometric measurements and assessment of the BBF were carried out 7 days after the last administration of asiatic acid. The bladder catheter was connected *via* a three-way stopcock to a pressure transducer (FT03; Grass Instruments) and to a microinjection pump (CMA 100; Microject, Solna, Sweden). Conscious cystometry was performed as previously described (Wróbel et al., 2016; Wróbel et al., 2018), by slowly filling the bladder with physiological saline at a constant rate of 0.05 ml/min to elicit repetitive voiding. Micturition volumes were measured by fluid collector attached to a force displacement transducer (FT03C; Grass Instruments). The following parameters, which clinical meaning had been explained by Andersson et al. (2011), were recorded: amplitude of nonvoiding contractions (ANVC; cm H_2_O), area under the pressure curve (AUC; cm H_2_O/sec), bladder compliance (BC; ml/cm H_2_O), basal pressure (BP; cm H_2_O), detrusor overactivity index (DOI; cm H_2_O/ml), frequency of nonvoiding contractions (FNVC; times/filling phase), intercontraction interval (ICI; s), micturition voiding pressure (MVP; cm H_2_O), postvoid residual (PVR; ml), threshold pressure (TP; cm H_2_O), volume threshold to elicit nonvoiding contractions (VTNVC; %), and voided volume (VV; ml). The measurements in each animal represent the average of five bladder micturition cycles after obtaining repetitive voiding.

The BBF was assessed by the laser Doppler blood perfusion imager (PeriScan PIM III, Perimed) and it was showed as changes in the laser doppler frequency by means of the color scale. The BBF was measured 5 times in each rat, immediately after emptying its bladder.

### Biochemical Analyses

After the cystometric procedures and assessment of the BBF the animals were killed by decapitation. Their urinary bladders were collected. Urothelium thickness and levels of the following parameters were measured in the bladder urothelium: 3-nitrotyrosine (3-NIT, LifeSpan BioSciences; CN LS-F40120–1), calcitonin gene related peptide (CGRP; Biomatik, CN EKU02858), E-Cadherin (CDH1; Abbexa Ltd, abx052816), interleukin 1β (IL-1β; Cloud-Clone, SEA563Ra), interleukin 6 (IL-6; LifeSpan BioSciences, LS-F25921-1), malondialdehyde (MAL, Biomatik, CN EKF57996), nitric oxide synthase 2, inducible (NOS2; Biomatik, EKU06310), organic cation transporter 3 (OCT3; Antibodies-online, CN ABIN6227163), substance P (SP; Biomatik, EKC40433), synaptic vesicle glycoprotein 2A (SV2A; MyBioSource; MBS9348576), synaptosomal-associated protein 23 (SNAP-23; MyBioSource, MBS9317604), synaptosomal-associated protein 25 (SNAP-25; Biomatik, EKF58391), tight junction protein 1 (ZO1; CUSABIO, CSB-E17287r), tumor necrosis factor α (TNF-α; LifeSpan BioSciences, LS-F5193), vascular endothelial growth factor A (VEGFA; LifeSpan BioSciences, LS-F2569). Concentrations of the vesicular acetylcholine transporter (VAChT; LifeSpan BioSciences, CN LS-F12924-1) were measured in the detrusor muscle. Additionally, urine levels of the brain-derived neurotrophic factor (BDNF; PROMEGA, CN G7610) and the nerve growth factor (NGF; LifeSpanBioSciences, CN LS-F25946-1) were determined. The measurements were carried out according to the manufacturers’ instructions. The outcomes were given in pg/ml. Each sample was analyzed in duplicate.

### Urothelium Thickness Measurement

The measurement of urothelium thickness was carried out in a blinded fashion as we described before (Wróbel et al., 2017). The bladder wall sagittal sections from the dome to the trigone were cut and stained with hematoxylin and eosin. The image analyzer computer system Leica Qwin 500 Image Analyzer (Leica Imaging Systems Ltd., Cambridge, England) was used to evaluate the urothelium thickness in micrometer using the interactive measure menu and hematoxylin and eosin-stained sections. A mean of 15 readings was estimated from five serial sections from slides of each animal in each group using low magnification (×10).

### Statistical Analysis

Two-way analysis of variance (ANOVA) followed by the Bonferroni’s post hoc test was applied for statistical calculations. The outcomes were given as the means ± standard deviation (SD), *p* < 0.05 was considered as statistically significant with 95% confidence. Due to the exploratory nature of the research, the obtained data are qualitative ones.

## Results

### Cystometric Analyses

An acute intravesical dose of 0.75% retinyl acetate solution produced significant alterations in most of the analyzed cystometric parameters. As summarized in Table 1, rats pretreated with retinyl acetate presented increased values of ANVC (by ca. 180% vs. the control group), AUC (by ca. 70%), BP (by ca. 60%), DOI (by ca. 210%), FNVC (by ca. 780%) and decreased values of BC (by ca. 30%), ICI (by ca. 30%), TP (by ca. 40%), VTNVC (by ca. 30%), VV (by ca. 35%). Only MVP and PVR remained at the control levels. The 14-day administration of asiatic acid did not affect cystometric parameters in the vehicle-pretreated animals, but it significantly improved the bladder condition of rats exposed to retinyl acetate. 30 mg/kg/day of asiatic acid was potent enough to normalize BC (two-way ANOVA: F(1,56) = 6.79; *p* = 0.0117 for the retinyl acetate-asiatic acid interaction), BP (F(1,56) = 7.40; *p* = 0.0087), DOI (F(1,56) = 51.27; *p* < 0.0001), ICI (F(1,56) = 17.29; *p* = 0.0001), TP (F (1,56) = 42.25; *p* < 0.0001), VTNVC (F(1,56) = 6.23; *p* = 0.0156), and VV (F(1,56) = 17.77; *p* < 0.0001) values. Moreover, it prevented elevation of ANVC (two-way ANOVA: F(1,56) = 24.34; *p* < 0.0001), AUC (two-way ANOVA: F(1,56) = 17.01; *p* = 0.0001), and FNVC levels (two-way ANOVA: F(1,56) = 29.21; *p* < 0.0001).

**TABLE 1:** Effects of the 14-days administration of asiatic acid (30 mg/kg/day, by oral gavage) on the cystometric parameters in conscious rats subjected to retinyl acetate therapy.

Substances	Amplitude of nonvoiding contractions (cm H_2_O)	Area under the pressure curve (cm H_2_O/sec)	Bladder compliance (ml/cm H_2_O)	Basal pressure (cm H_2_O)	Detrusor overactivity index (cm H_2_O/ml)	Frequency of nonvoiding contractions (times/filling phase)	Intercontraction interval (s)	Micturition voiding pressure (cm H_2_O)	Postvoid residual (ml)	Threshold pressure (cm H_2_O)	Volume threshold to elicit nonvoiding contractions (%)	Voided volume (ml)
Vehicle (acute) +vehicle (14 days)	2.269 ± 0.159	12.333 ± 1.877	0.220 ± 0.0390	3.027 ± 0.7459	74.667 ± 15.59	0.549 ± 0.1693	1,018 ± 142.0	42.427 ± 9.272	0.071 ± 0.0181	7.673 ± 1.277	81.133 ± 7.605	0.849 ± 0.1993
RA (acute) +vehicle (14 days)	6.285 ± 2.021***	20.667 ± 3.266***	0.149 ± 0.0228***	4.753 ± 0.9561***	229.200 ± 57.46***	4.834 ± 1.775***	703.20 ± 131.6***	35.807 ± 10.97	0.073 ± 0.0143	4.380 ± 1.301***	58.400 ± 10.80***	0.547 ± 0.1401**
Vehicle (acute) + AA (14 days)	2.283 ± 0.1816	12.533 ± 1.959	0.224 ± 0.0547	2.573 ± 0.6850	70.067 ± 27.32	0.702 ± 0.1697	895.6 ± 153.3	41.387 ± 10.11	0.063 ± 0.0203	6.727 ± 1.127	87.067 ± 7.824	0.732 ± 0.2041
RA (acute) + AA (14 days)	3.461 ± 0.8983**^^^	15.667 ± 2.410***^^^	0.203 ± 0.0228^^^	3.140 ± 0.8879^^^	101.067 ± 13.20^^^	2.197 ± 0.8863***^^^	911.87 ± 184.7^^^	40.433 ± 10.68	0.077 ± 0.0145	8.387 ± 2.029^^^	76.533 ± 11.08^^^	0.957 ± 0.3656^^^

Retinyl acetate (RA, 0.75% solution in polysorbate 80 in saline) was given intravesically as an acute administration and asiatic acid (AA, 30 mg/kg/day) was administered by oral gavage for 14 days. Cystometric evaluation was performed 7 days after the last administration of asiatic acid. The values represent the mean ± SD (n = 15 rats per group). The obtained data were assessed by the two-way analysis of variance (ANOVA) followed by Bonferroni’s *post hoc* test. **p < 0.01, ***p < 0.001 vs. vehicle-treated group; ^ ^ ^ p < 0.001 vs. RA-treated group.

### BBF and Urothelium Thickness

**FIGURE 1:**
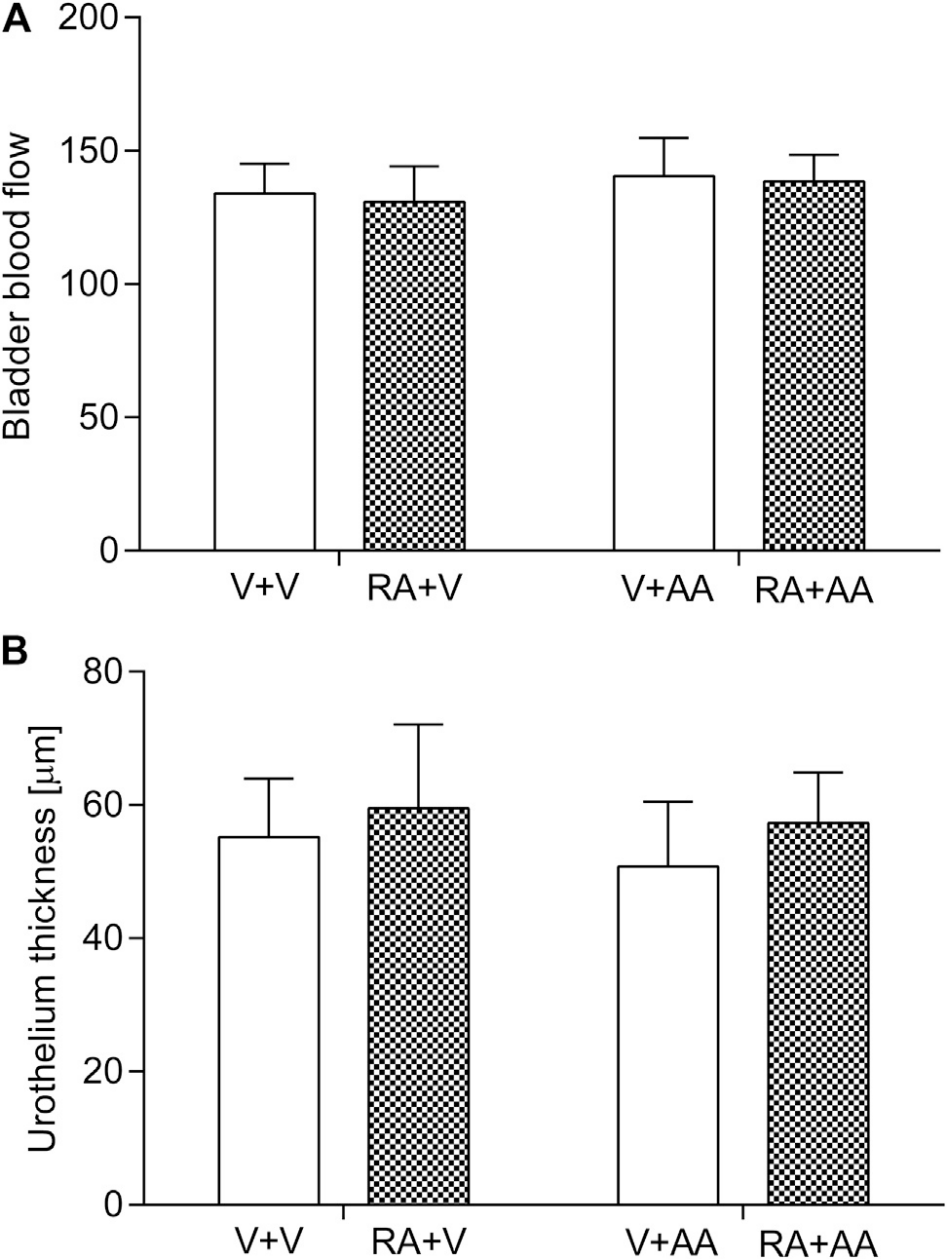
Influence of the 14-day administration of asiatic acid (AA, 30 mg/kg/day, oral gavage) on the **(A)** bladder blood flow and **(B)** urothelium thickness in rats subjected to the retinyl acetate intravesical treatment (RA, 0.75% solution in polysorbate 80 in saline). The values represent the mean + SD (n = 15 animals per group). V–Vehicle.

Neither acute intravesical instillation of retinyl acetate nor treatment with asiatic acid (30 mg/kg daily for 14 days) influenced the BBF or urothelium thickness of the tested animals (*p* > 0.05). The results were shown in Figure 1. Consequently, two-way ANOVA did not detect any significant interaction between retinyl acetate and asiatic acid in relation to the BBF analysis: F(1,56) = 0.04; *p* = 0.8521 or to the urothelium thickness analysis: F(1,56) = 0.19; *p* = 0.6672.

### Biochemical Study

The 14-day treatment with asiatic acid (30 mg/kg/day) given by the oral gavage did not influence the assessed biochemical parameters in the vehicle-pretreated rats, except for the VAChT, OCT3, and SVA2 levels. The asiatic acid therapy significantly diminished VAChT, OCT3, and SVA2 concentrations by ca. 30%, 45%, and 20% vs. the control group, respectively.

#### Calcitonin Gene Related Peptide, Tight Junction Protein 1, Substance P, E-Cadherin Levels in the Bladder Urothelium

**FIGURE 2:**
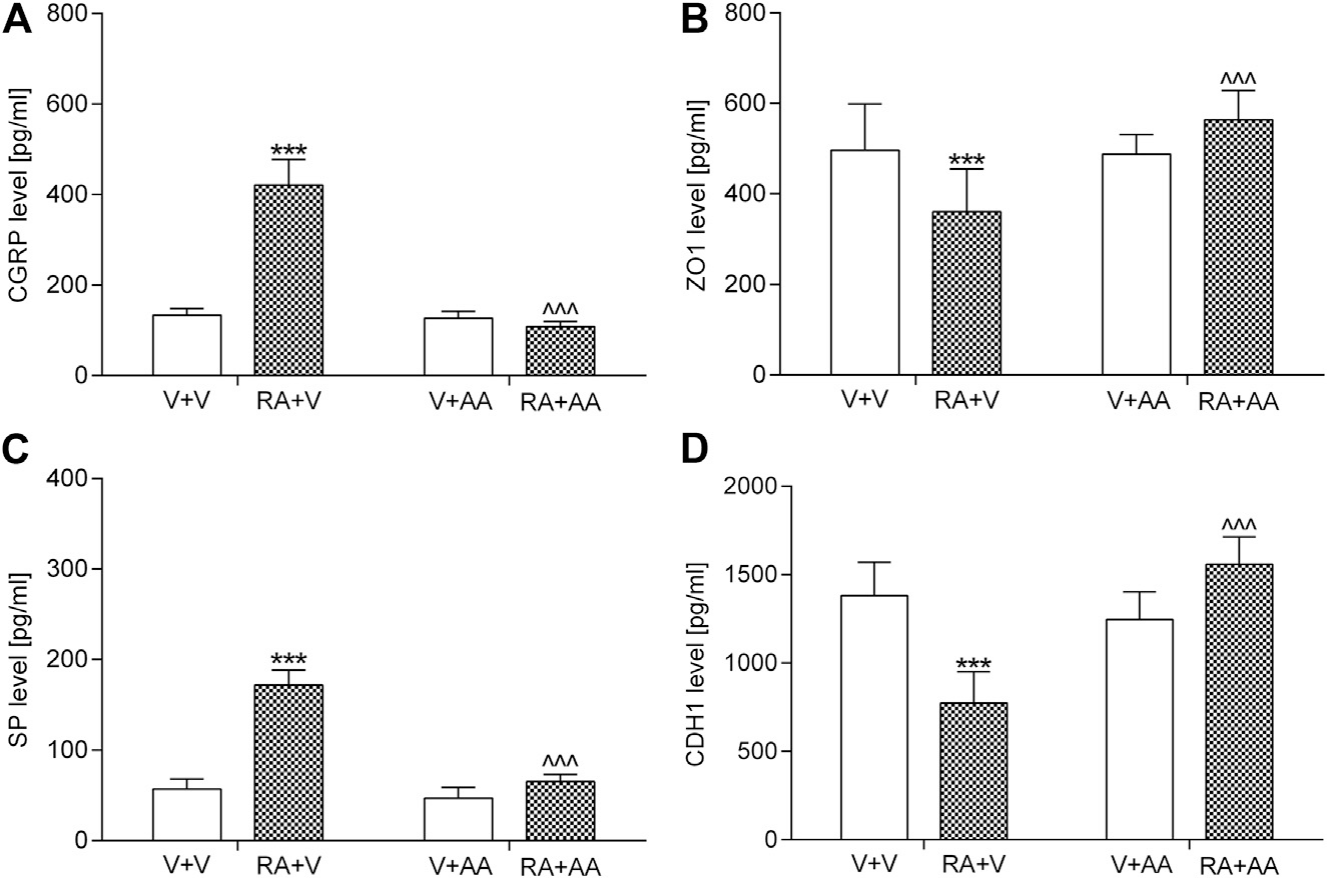
Influence of the 14-day administration of asiatic acid (AA, 30 mg/kg/day, oral gavage) on **(A)** calcitonin gene related peptide (CGRP) **(B)** tight junction protein 1 (ZO1) **(C)** substance P (SP), and **(D)** E-Cadherin (CDH1) in the urothelium in rats subjected to the retinyl acetate intravesical treatment (RA, 0.75% solution in polysorbate 80 in saline). The values represent the mean + SD (n = 15 animals per group). ***p < 0.001 vs. vehicle-treated group (V + V), ^ ^ ^ *p* < 0.001 vs. retinyl acetate-treated group (Bonferroni’s post hoc test).

An acute intravesical instillation of 0.75% solution of retinyl acetate significantly elevated CGRP (by ca. 215%) and SP (by ca. 200%) values as well as reduced CDH1 (by ca. 40%) and ZO1 (by ca. 30%) concentrations in the tested samples (Figure 2). The 14-day administration of asiatic acid (30 mg/kg/day) prevented the above-mentioned biochemical changes. Statistical calculations (two-way ANOVA) indicated significant retinyl acetate-asiatic acid interactions for the analysis of CGRP (F (1,56) = 371.89; *p* < 0.0001), ZO1 (F (1,56) = 26.13; *p* < 0.0001), SP (F (1,56) = 221.90; *p* < 0.0001), and CDH1 (F (1,56) = 109.30; *p* < 0.0001).

#### Organic Cation Transporter 3, Synaptosomal-Associated Protein 23, Synaptosomal-Associated Protein 25, SVA2 Levels in the Bladder Urothelium and VAChT Levels in the Bladder Detrusor Muscle

**FIGURE 3:**
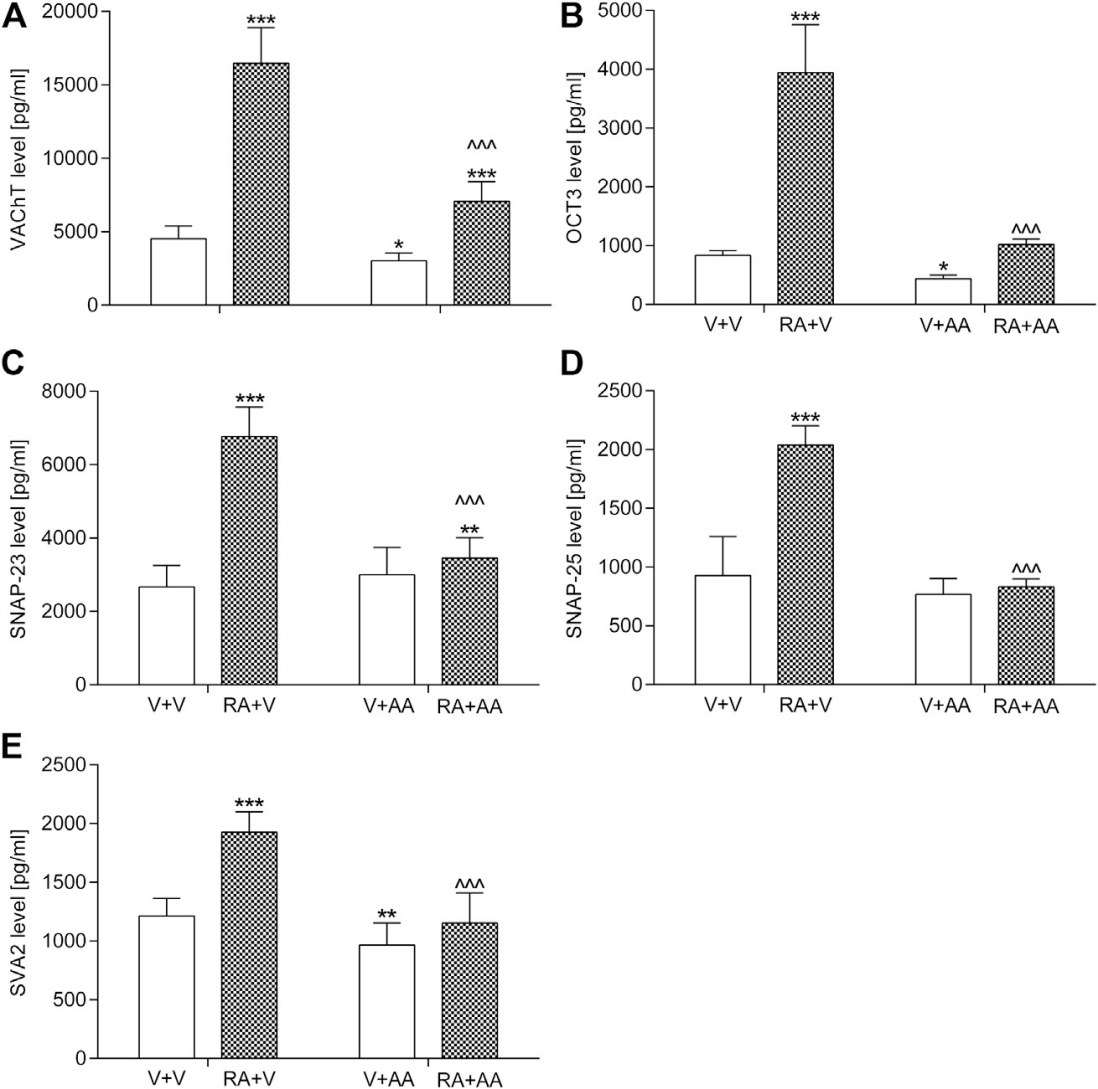
Influence of the 14-day administration of asiatic acid (AA, 30 mg/kg/day, oral gavage) on **(A)** the levels of vesicular acetylcholine transporter (VAChT) in the bladder detrusor muscle and levels of **(B)** organic cation transporter 3 (OCT3), **(C)** synaptosomal-associated protein 23 (SNAP-23), **(D)** synaptosomal-associated protein 25 (SNAP-25), and **(E)** synaptic vesicle glycoprotein 2A (SV2A) in the urothelium in rats subjected to the retinyl acetate intravesical treatment (RA, 0.75% solution in polysorbate 80 in saline). The values represent the mean + SD (n = 15 animals per group). ***p < 0.001, **p < 0.01, *p < 0.05 vs. vehicle-treated group (V + V), ^ ^ ^ *p* < 0.001 vs. retinyl acetate-treated group (Bonferroni’s post hoc test).

As presented in Figure 3, animals exposed to the intravesical instillation of 0.75% solution of retinyl acetate had elevated levels of OCT3 (by ca. 370% vs. the control group), SNAP-23 (by ca. 150%), SNAP-25 (by ca. 120%), and SVA2 (by ca. 60%) in the bladder urothelium and VAChT levels (by ca. 260%) in the bladder detrusor muscle. The 2-week treatment with asiatic acid (30 mg/kg/day, by the oral gavage) prevented an increase in protein values. However, only OCT3, SNAP-25, and SVA2 levels had the basal values, whereas SNAP-23 and VAChT remained higher (by ca. 30% and 60%, respectively). Statistical calculations indicated significant retinyl acetate-asiatic acid interactions for the analysis of OCT3 (F(1,56) = 139.83; *p* < 0.0001), SNAP-23 (F(1,56) = 106.72; *p* < 0.0001), SNAP-25 (F(1,56) = 103.37; *p* < 0.0001), SVA2 (F(1,56) = 27.04; *p* < 0.0001), and VAChT (F(1,56) = 107.87; *p* < 0.0001).

#### IL-1β, IL-6, TNF-α, Malondialdehyde, 3-Nitrotyrosine, Nitric Oxide Synthase 2, Vascular Endothelial Growth Factor A Levels in the Bladder Urothelium and Brain-Derived Neurotrophic Factor and NGF Levels in Urine

**FIGURE 4:**
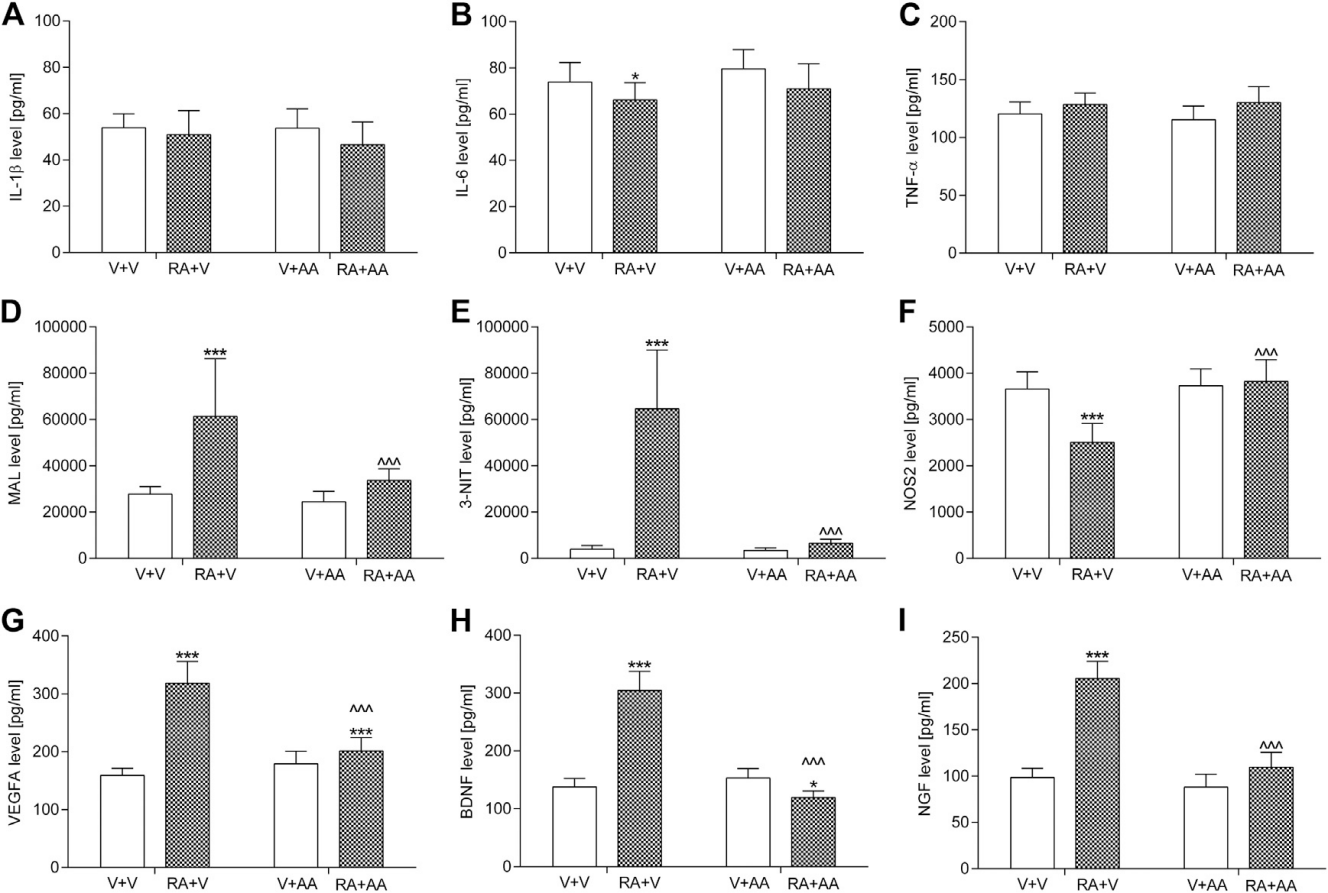
Influence of the 14-day administration of asiatic acid (AA, 30 mg/kg/day, oral gavage) on urothelial levels of **(A)** interleukin 1β (IL-1β) **(B)** interleukin 6 (IL-6) **(C)** tumor necrosis factor α (TNF-α) **(D)** malondialdehyde (MAL) **(E)** 3-nitrotyrosine (3-NIT), and **(F)** nitric oxide synthase 2 (NOS2) **(G)** vascular endothelial growth factor A (VEGFA) in the urothelium as well as urine levels of **(H)** brain-derived neurotrophic factor (BDNF) and **(I)** nerve growth factor (NGF) in rats subjected to the retinyl acetate intravesical treatment (RA, 0.75% solution in polysorbate 80 in saline). The values represent the mean + SD (n = 15 animals per group). ***p < 0.001, *p < 0.05 vs. vehicle-treated group (V + V), ^ ^ ^*p* < 0.001 vs. retinyl acetate-treated group (Bonferroni’s post hoc test).

Exposition to an acute intravesical dose of 0.75% solution of retinyl acetate generally did not result in a considerable elevation of the proinflammatory cytokines IL-1β and TNF-α in the bladder urothelium when compared to the control animals, but it slightly decreased IL-6 levels. On the other hand, such a pretreatment increased MAL (by ca. 120%), 3-NIT (by ca. 1,500%), VEGFA (by ca. 100%), BDNF (by ca. 120%), and NGF (by ca. 110%) levels in the tested samples as well as it reduced NOS2 values (by ca. 30%) in the bladder urothelium. Administration of asiatic acid (30 mg/kg/day for 14 days) prevented the above-mentioned changes. The parameters were within basal levels except for the VEGFA concentration which was partially elevated (by ca. 30% vs. the control group). The outcomes were illustrated in Figure 4. Two-way ANOVA showed a significant retinyl acetate-asiatic acid interaction for the analysis of MAL: F(1,56) = 13.21; *p* = 0.0006, 3-NIT: F(1,56) = 76.07; *p* < 0.0001, NOS2: F(1,56) = 35.50; *p* < 0.0001, VEGFA: F(1,56) = 108.35; *p* < 0.0001, BDNF: F(1,56) = 349.02; *p* < 0.0001, and NGF: F(1,56) = 122.58; *p* < 0.0001.

## Discussion

As far as we know, this is the first study investigating the possibility of using asiatic acid in the prevention (and/or treatment) of OAB and the first report of beneficial effects of this natural compound in an animal model of DO. Asiatic acid given daily *via* oral gavage for 14 days at a dose of 30 mg/kg prevented the pathological changes in the involuntary bladder behavior observed in female Wistar rats exposed to an intravesical administration of retinyl acetate. This activity was measured by standard urodynamic pressure-volume parameters. Moreover, asiatic acid normalized levels of several markers indicating deterioration of the urinary bladder function in the tested animals.

Cystometric measurements belong to diagnostic procedures of OAB. In animals pretreated with retinyl acetate instillation, treatment with asiatic acid shared the activity profile with oxibutynin chloride (0.5 mg/kg, intravenously) (Wróbel et al., 2015). In our previous experiments (Wróbel et al., 2015) we confirmed that the retinyl acetate-induced rat model of DO responds to antimuscarinic treatment. A single intravenous dose of oxybutynin chloride (0.5 mg/kg) reversed the changes of cystometric parameters caused by administration of 0.75% retinyl acetate solution. It decreased the elevated levels of BP, TP, MVP, DOI, ANVC, and FNVC as well as it increased the reduced levels of VV, PVR, ICI, BC, VTNVC. We guess that restoration of the DOI value is the best proof of asiatic acid effectiveness in the applied model of DO. Following Miller et al. (2002), functional bladder capacity is a reliable indicator of DO grade, and DOI is defined as the quotient of the sum of amplitudes of all detrusor contractions during the filling phase and functional bladder capacity (Wróbel et al., 2019). Attenuation of bladder hyperactivity was also confirmed by reduction of the increased amplitude and frequency of non-voiding contractions (NVC). Consequently, the volume threshold of the urinary bladder at which NVC appears was elevated. NVC can be treated as a comparable parameter to involuntary contractions in humans during the filling phase which are usually intensified in patients with DO. VTNVC in rodent cystometry is considered as an analogue to the volume at first involuntary detrusor contraction in humans (Behr-Roussel et al., 2012). Additionally, in animals pretreated with retinyl acetate the therapy with asiatic acid prevented bladder distensibility (measured by BC) (Harding et al., 2009), basal tension of the bladder (BP), bladder pressure immediately before micturition (TP) as well as AUC and ICI values. It should be emphasized that the natural compound tested in our study did not influence cystometric parameters in healthy rats and that it maintained urine storage without impairment of the voiding function measured by MVP and PVR. Furthermore, our results were in compliance with the general assumption that bladder emptying is adequate when the PVR value is less than 1/3 of the VV value (Dagdeviren et al., 2017). Elevated PVR is an indicator of voiding dysfunction. In fact, neither group of rats tested in our study presented increased levels of PVR, but Dağdeviren et al. (2017) reported that such an abnormality is not always observed in DO.

Outcomes of multiple pre-clinical and clinical studies suggest an existence of association between the pathogenesis of OAB and oxidative stress (Speich et al., 2020), activation of immune system, and increased urinary secretion of neurotrophins (Bhide et al., 2013), which was partially also recorded in our experiments. Elevated values of MAL, 3-NIT, IL-1β, IL-6, and TNF-α were observed in patients and laboratory animals with signs of OAB and bladder inflammation (Gonzalez et al., 2015; Ding et al., 2017; Bae et al., 2019; Barut et al., 2019). Raised urinary NGF and BDNF values were detected in patients with DO (Frias et al., 2011) and in OAB patients (Antunes-Lopes and Cruz, 2019). In our experiments, administration of 0.75% retinyl acetate solution induced changes in urothelial levels of MAL, 3-NIT, and NOS2 which indicated an oxidative damage in the bladder urothelium (Gonzalez et al., 2015; Barut et al., 2019) but it did not induce urothelial inflammation–neither the urothelium thickness nor urothelial levels of the pro-inflammatory cytokines (IL-1β, IL-6, TNF-α) were considerably affected. However, both BDNF and NGF concentrations in urine of the retinyl acetate-exposed animals were significantly higher than those detected in urine of the control group. Treatment with asiatic acid prevented oxidative damage of the urothelium and normalized urinary secretion of the tested neurotrophins. The anti-inflammatory and antioxidant activity of asiatic acid have been found before (Dong et al., 2017; Qi et al., 2017). However, the present studies revealed that these effects should be rather expected in subjects with ongoing inflammation or with elevated levels of oxidative stress markers than in healthy subjects. Basing on the literature data (Antunes-Lopes et al., 2011), we guess that our outcomes related to the impact of asiatic acid on the urinary levels of BDNF and NGF are of a particular clinical significance. More and more scientists consider BDNF and NGF values in urine as reliable tools to monitor the therapeutic effect of drugs used in the pharmacotherapy of OAB. BDNF and NGF are secreted urothelial and detrusor smooth muscles. Most probably, elevated levels of NGF in urine could sensitize the afferent pathways in the bladder, enhance bladder sensory input which ultimately may cause development of DO. In fact, patients with OAB presented high urinary concentration of NGF. Similarly, BDNF contributes to the normal functioning of sensory neurons, and urinary BDNF level is suggested as a potential biomarker of OAB. Regarding the molecular mechanism of the retinyl acetate induction of DO, it is worth mentioning that TRPV_1_ are essential downstream receptors for NGF effects in the bladder. Both antimuscarinic agents and botulinum toxin, similarly to asiatic acid, diminish the urinary levels of BDNF and NGF in patients with bladder overactivity. Thus, our findings should encourage further studies on asiatic acid as a promising agent for the management of this condition.

Animals exposed to the intravesical instillation of retinyl acetate presented abnormalities in urothelial/detrusor values of several further biomarkers that are involved in the functioning of the urinary bladder. In the tested samples we found reduced levels of proteins responsible for the urothelial barrier (i.e., ZO1 and CDH1). ZO1 belongs to cytoplasmic proteins of tight junctions, whereas CDH1 is one of the adhesive molecules of adherens junctions that maintain the integrity of epithelial tissues. Reduction in ZO1 and CDH1 can cause ruptures in cell to cell contacts as well as an increase in bladder urothelial permeability which results in passage of small ions across the blood–urine barrier. Significantly lower ZO1 and CDH1 levels were noted in patients with urothelial dysfunctions (Liu et al., 2012; Lee and Lee, 2014). Furthermore, in the retinyl acetate-exposed rats we detected increased urothelial concentrations of two crucial neurotransmitters of bladder afferents–CGRP and SP. These neuropeptides co-regulate smooth muscle contractions and are secreted in response to harmful stimuli, playing a significant role in the inflammatory process (Smet et al., 1997). Retinyl acetate-treated rats had also elevated urothelial levels of VEGFA that is a well-known modulator of the micturition reflex pathways that mediates inflammation in the urinary bladder and influences the density of cholinergic nerve fibers in urothelial layers and the detrusor muscle. VEGF levels are elevated patients with painful bladder syndrome. It modifies bladder function (i.e., decreases duration of intermicturition interval, reduces voiding pressure, micturition volume, and bladder capacity) and visceral sensitivity. There are suggestions that VEGF might change the permeability of the urothelium *via* mechanisms similar to the ones involved in vascular permeability. Moreover, there is some evidence that this growth factor, similarly to NGF and BDNF, may be implicated in bladder neuroplasticity (Malykhina et al., 2012). We also found out that animals subjected to retinyl acetate instillation presented higher levels of proteins involved in acetylcholine transportation (i.e., VAChT and OCT3) (Lips et al., 2007), and release of various transmitters, including acetylcholine (SNAP-23, SNAP-25, SVA2) (Hanna-Mitchell et al., 2015; Bartholome et al., 2017). Our observations are generally in line with results from other pre-clinical experiments and reports from clinical trials. We previously had demonstrated that spontaneously hypertensive female Wistar-Kyoto rats with DO presented the same changes in CGRP, OCT3, and VAChT levels (Wróbel et al., 2020). Smet et al. (1997) found an increased distribution of specific nerve fibers containing CGRP and SP in the urinary bladder of patients with detrusor instability, and Tamaki et al. (2004) reported that subjects with interstitial cystitis may present overexpression of VEGFA in the urinary bladder. On the other hand, though the animals exposed to retinyl acetate in our study manifested a significant impairment of the urothelium barrier function, Liu et al. (2012) did not detect such an anomaly in patients with OAB.

The outcomes of the present work revealed that the 14-day oral administration of asiatic acid at a dose 30 mg/kg/day maintained functions of urinary bladders of animals with the induced DO. Such a therapy prevented all of the above-mentioned biochemical changes in both detrusor muscle and bladder urothelium. Our findings related to the beneficial impact of asiatic acid on smooth muscle contractions, transportation and release of transmitters (including acetylcholine), and functioning of the urothelial barrier are a completely new issue. It’s worth mentioning that the activity of asiatic acid toward the urinary bladder is similar to the one observed for botulinum toxin A. A range of experiments have revealed that botulinum toxin A inhibits release of CGRP, SP (Rapp et al., 2006), reduces expression of SNAP-23, SNAP-25 (Hanna-Mitchell et al., 2015), diminishes the number of cholinergic VAChT-positive nerve terminals (Lepiarczyk et al., 2011) in animal models with urinary bladder dysfunctions. In patients with bladder conditions injection of onabotulinumtoxin A decreased VEGF levels (Peng et al., 2013) and increased ZO1 and CDH1 values (Chen et al., 2016) in the bladder tissue.

Though the molecular mechanisms of the observed beneficial effects of asiatic acid toward the urinary bladder functions were not a subject of the present study, we can assume that they are due to an interplay between this compound and several enzymes, growth and transcription factors as well as different cell signaling cascades. Amongst them, acetylcholinesterase, cyclooxygenase-2, GABA_B_ receptors, SVA2 and SNAP25 proteins, JAK1/STAT3, NF-κB, RhoA/ROCK, ERK1/2, and NO-dependent signaling pathways should be mentioned (for review see Nagoor Meeran et al., 2018). Furthermore, since asiatic acid prevents the retinyl acetate-induced DO, it is possible that this compound somehow interacts with/blocks TRPV_1_ receptors. It has been demonstrated that stimulation of TRPV_1_ receptors by retinyl acetate is responsible for evoked symptoms of DO in animals (Asfaw et al., 2011; Wróbel et al., 2015). TRPV_1_ receptors also known as ionotropic receptors for retinoids are situated on afferent neurons (type C and type Aδ) and they activate nociceptive sensory neurons and mediate sensory hypersensitivity. Shijin et al. (2013) found out that selective TRPV_1_ antagonists could be beneficial for treating retinoid-induced sensory hypersensitivity. Thus, asiatic acid could influence the TRPV_1_ receptor-dependent pathways as well and *via* this mechanism prevent instability of the urinary bladder detrusor. However, it is only a speculative opinion that should be verified by appropriate *in vitro* and *in vivo* studies.

Based on the outcomes of the present project as well as on investigational data of other authors cited above, asiatic acid has emerged as a very promising natural compound that could be useful in the management of various diseases, including urinary bladder conditions with DO. In fact, the traditional (mostly topical) use of *C. asiatica* preparations containing asiatic acid dates back to more than 30 years ago. However, so far little clinical data is accessible on its bioavailability, efficiency, and safety profile after oral administration. In their latest clinical studies, Songvut et al. (2019) found out that the pharmacokinetics of the standardized extract of *C. asiatica* given to healthy volunteers in a form of capsules was significantly different from the one observed in animal experiments. However, the extract was safe and well-tolerated after single and repeated administration at both tested doses, i.e., 250 and 500 mg. In other clinical studies (Incandela et al., 2001; Lou et al., 2018) the preparation of the triterpenic fraction of *C. asiatica* containing 23% of asiatic acid was well-tolerated up to 240 mg when taken orally for one year.

We admit that our current study has some limitations such as the lack of male groups for comparison to female subjects. According to the literature data (Andersson et al., 2011), there are some differences between micturition in female and male rats. In our lab we had designed two animal models of DO, i.e. the corticosterone-induced model (Wróbel et al., 2016) and the retinyl acetate-induced one (Wróbel et al., 2015), both of which were confirmed in female Wistar rats. Female sex is considered as more prone to the development of OAB, though this condition is also quite frequently diagnosed in men. However, further studies on a reliable and reproducible male animal model of OAB should be performed to check whether the effects of asiatic acid on the urodynamic parameters and bladder functioning are/are not gender-dependent. And, certainly, not all parameters recorded during rodent cystometry can be directly extrapolated to human population (Andersson et al., 2011). Therefore, all results of the pre-clinical studies have to be confirmed in clinical trials before drawing far-reaching conclusions.

## Conclusion

A far as we know, this is the first report of beneficial effects of asiatic acid on the micturition cycle in an animal model of DO in female Wistar rats exposed to retinyl acetate instillation. In our opinion, the following results of current project should be emphasized: 1) asiatic acid given by oral gavage normalizes the cystometric parameters corresponding to DO and prevents the accompanying oxidative stress and increased urinary secretion of neurotrophins but it does not influence these parameters in healthy subjects, 2) asiatic acid prevents the changes in a range of biomarkers indicating a dysfunction of the urinary bladder, associated with smooth muscle contractions, urothelial barrier, or transportation and release of transmitters.

Multiple pre-clinical data and scarce clinical data encourage the notion that asiatic acid could have potential therapeutic value in the management of several chronic diseases (e.g., diabetes, epilepsy, hypertension, osteoporosis, Alzheimer disease, or cancer). Results of our present project contribute to these findings, suggesting the possible role of this natural compound in the prevention (and/or, speculatively, treatment) of conditions accompanied by DO, such as OAB. However, further studies are necessary.

## Data Availability Statement

The raw data supporting the conclusions of this article will be made available by the authors, without undue reservation.

## Ethics Statement

The animal study was reviewed and approved by The Local Ethics Committee in Lublin.

## Author Contributions

Conceptualization–AW; Investigation–AW, AnS, AlS, EP; Data Analysis–AW, AS; Writing (Original Draft Preparation–AnS; Writing (Review and Editing)–AW, AlS; Supervision–AW, EP; Funding Acquisition–AW.

## Funding

This study was supported by Funds for Statutory Activity of Medical University of Lublin, Poland.

## Conflict of Interest

The authors declare that the research was conducted in the absence of any commercial or financial relationships that could be construed as a potential conflict of interest.

## Abbreviations

3-NIT, 3-nitrotyrosine; ANVC, amplitude of nonvoiding contractions; AUC, area under the pressure curve; BP, basal pressure; BBF, bladder blood flow; BC, bladder compliance; BDNF, brain-derived neurotrophic factor; CGRP, calcitonin gene related peptide; DO, detrusor overactivity; DOI, detrusor overactive index; CDH1, E-Cadherin; FNVC, frequency of nonvoiding contractions; ICI, intercontraction interval; MAL, malondialdehyde; MVP, micturition voiding pressure; NGF, nerve growth factor; NOS2, nitric oxide synthase 2 (inducible); OCT3, organic cation transporter 3; PVR, postvoid residual; SP, substance P; SV2A, synaptic vesicle glycoprotein 2A; SNAP-23, synaptosomal-associated protein 23; SNAP-25, synaptosomal-associated protein 25; ZO1, tight junction protein 1; TP, threshold pressure; VEGFA, vascular endothelial growth factor A; VAChT, vesicular acetylcholine transporter; VV, voided volume; VTNVC, volume threshold to elicit nonvoiding contractions.
